# The Impact of Digital Technologies in Shaping Weight Loss Motivation Among Children and Adolescents

**DOI:** 10.3390/children12060685

**Published:** 2025-05-26

**Authors:** Małgorzata Wąsacz, Izabela Sarzyńska, Joanna Błajda, Natasza Orlov, Marta Kopańska

**Affiliations:** 1 Department of Medical Psychology, Faculty of Medicine, University of Rzeszów, 35-959 Rzeszow, Poland; mwasacz@ur.edu.pl (M.W.); norlov@ur.edu.pl (N.O.); 2 Student Research Club “Reh-Tech”, Faculty of Medicine, University of Rzeszów, 35-959 Rzeszow, Poland; sarzynskaizabela-2001@wp.pl; 3 Faculty of Health Sciences and Psychology, University of Rzeszów, 35-959 Rzeszow, Poland; jblajda@ur.edu.pl

**Keywords:** childhood obesity, digital technologies, motivation, BMI reduction, mobile apps, health

## Abstract

Background/Aim: Child and adolescent obesity is currently one of the most pressing public health challenges. Digital technology-based interventions are becoming increasingly important in supporting weight loss motivation and promoting healthy lifestyles. This review aims to assess the effectiveness of technology tools on the BMI (body mass index) and their impact on health attitudes in children and adolescents. Materials and Methods: The study was conducted according to PRISMA guidelines, analysing studies published between 2011 and 2024 on PubMed, Scopus, Web of Science and Google Scholar databases. Of the 1475 articles identified and analysed, 59 met the inclusion criteria. Studies were assessed based on the type of technology used, the type of intervention, family involvement, the level of personalisation and their impact on BMI and motivation. Results: The systematic review showed that digital technologies—in particular mobile apps, wearables and m-health platforms—can effectively support weight reduction and improved eating habits in children and adolescents. The most beneficial results were observed in interventions that were personalised and included caregiver support. In addition, digital technology was shown to have a positive impact on participants’ psychological well-being. Conclusions: Digital technology-based interventions can be an effective tool in the prevention and treatment of obesity in children and adolescents. However, their success depends on a comprehensive approach that includes psychological, social and cognitive developmental factors.

## 1. Introduction

Obesity and overweight in children and adolescents represent one of the most serious global health challenges. Epidemiological data indicate that the number of children with excess body weight is steadily increasing, posing a growing public health threat worldwide. Overweight and obesity are defined as abnormal or excessive fat accumulation that poses a health risk [1]. In 2019, the World Obesity Federation estimated that by 2025, 206 million children and adolescents aged 5 to 19 years will be living with obesity, and this number is expected to rise to 254 million by 2030 [2]. Being overweight in childhood and adolescence is a significant risk factor for obesity in adulthood [3]. Beyond traditional strategies such as promoting a healthy diet and physical activity, growing attention is being paid to how digital technologies can support behavioural change and health motivation in young populations. These strategies should include a well-balanced diet, regular physical activity, adequate sleep hygiene and support from the proximal social environment. Such measures can contribute to long-term health improvement and the development of sustainable health-promoting habits [4].

Although this paper focuses on digital technologies that support weight loss motivation, it is important to note the growing role of Continuous Glucose Monitoring (CGM) systems in adolescents with type 2 diabetes. CGM allows for real-time glucose monitoring, which supports not only medical treatment but also enhances health awareness and behavioural self-regulation. CGM replaces blood glucose (BG) monitoring via traditional glucometers. The use of CGM—both in adults and children with type 2 diabetes—has been associated with achieving and maintaining target HbA1c levels, reducing severe hypoglycaemia, and increasing treatment satisfaction. Since obesity and type 2 diabetes often coexist in adolescents, CGM may serve as an additional motivational component in comprehensive health interventions [5].

One of the key tools used to assess the nutritional status of children and adolescents is the body mass index (BMI), calculated as the ratio of weight (kg) to the square of height (m^2^). In the paediatric population, the BMI is interpreted using age- and gender-specific percentile charts. A value above the 85th percentile indicates overweight, while a value above the 95th percentile indicates obesity. Persistently elevated BMI values in childhood increase the risk of being overweight in adulthood and developing serious metabolic conditions such as type 2 diabetes, atherosclerosis, hypertension and lipid disorders [6].

While several systematic reviews have investigated the general effectiveness of digital technologies in addressing childhood obesity, few have specifically examined their influence on motivation. Motivation plays a central role in determining whether children and adolescents adhere to healthy behaviours such as physical activity, dietary changes, or reduced screen time. Understanding how digital technology interventions can foster and maintain motivation is essential for designing more effective, long-lasting strategies for weight management in youth. To assess the outcomes of such interventions, anthropometric measures are commonly used. Among these, the BMI remains the most widely applied metric in paediatric obesity research due to its simplicity and accessibility [7].

**Figure 1:**
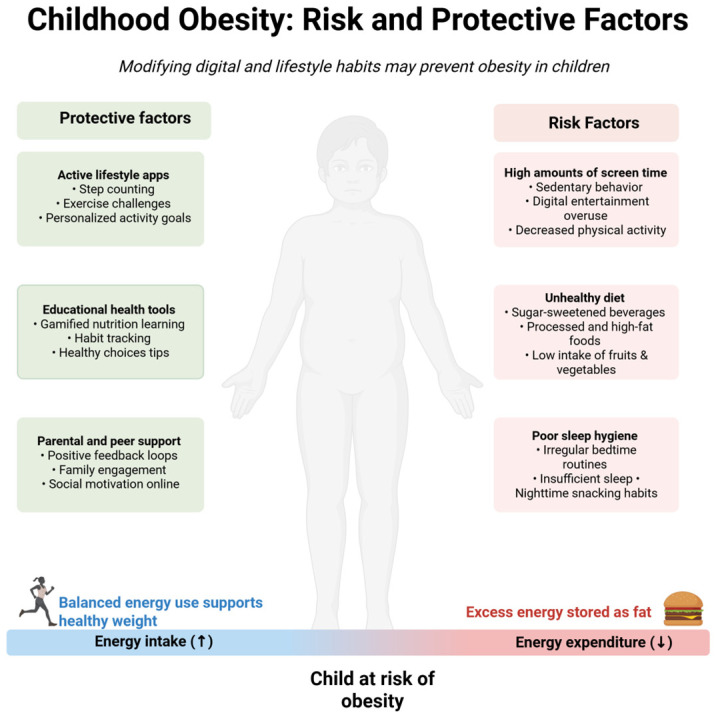
Obesity in children: risk and protective factors. Created with BioRender.com.

The prevalence of obesity continues to rise. The aetiology of obesity in children and adolescents is multifactorial and involves complex interactions between genetic, environmental, behavioural and psychosocial factors. Weight regulation shows considerable biological variability, with some individuals naturally maintaining healthy levels of body fat, while others strive to achieve energy balance throughout life. An important role in the development of obesity is played by the home environment, which shapes the occurrence of certain dietary patterns, such as a high-calorie diet, a lack of regular physical activity, disrupted sleep patterns, and excessive time spent in front of a screen [8]. Studies have shown that children from lower socio-economic backgrounds are significantly more likely to be overweight compared to peers from more affluent families [9]. Other important risk factors include maternal obesity before pregnancy, excessive weight gain during pregnancy and gestational diabetes [10]. Among the most common behaviours contributing to obesity is a high consumption of sugar-sweetened beverages and nutrient-poor foods high in saturated fat [11]. It is therefore important to implement modern digital technology solutions to minimise the risk of obesity by supporting healthy eating habits, promoting physical activity and raising health awareness among children and adolescents (Figure 1).

The rapid development of digital technologies has led to their increased use in preventive and therapeutic interventions targeting children and adolescents with excessive body weight. Digital tools—including mobile apps, eHealth platforms, educational games, Internet of Things (IoT) systems, wearable devices that monitor physical activity and artificial intelligence-based tools—offer new opportunities to support healthy lifestyles, weight management and improved eating habits [12]. These technologies allow for the personalisation of interventions, monitoring progress in real time, providing feedback and engaging users through gamification elements and social challenges. This significantly increases the motivation and engagement of children and young people in the weight loss process [13].

This review aims to fill the gap in the research by analysing how digital technologies influence weight loss motivation among children and adolescents. To our knowledge, this is the first systematic review that concentrates specifically on how digital technology interventions impact weight loss motivation—rather than general outcomes like the BMI or physical activity—among children and adolescents.

## 2. Materials and Methods

### 2.1. Search Strategy

The current review was conducted in line with the Preferred Reporting Items for Systematic Reviews and Meta-Analyses (PRISMA) guidelines for systematic reviews and meta-analyses [14].

The review protocol was pre-registered on the Open Science Framework prior to publication.

This review aims to clarify the current state of knowledge on the impact of digital technologies on weight loss motivation and health-related behaviours among children and adolescents. A comprehensive search of the literature was conducted in four databases: PubMed, Scopus, Web of Science and Google Scholar. The search used a combination of the following keywords: childhood obesity, weight loss motivation, mobile health, BMI reduction, school-aged children, mHealth, mobile apps, wearable devices, serious games, self-monitoring, physical activity. Keywords were combined using AND/OR logical operators and tailored to the specific requirements of each search engine.

### 2.2. Inclusion and Exclusion Criteria

Publications were included if they met the following criteria:

- They were published between 2011 and 2024;
- They were available in full-text format;
- Their research focus was on the population of children and adolescents of school age (6–18 years);
- The research assessed impact of digital technology on motivation to lose weight and change BMI was investigated;
- They were published in English.

Publications were excluded if they met the following criteria:

- They focused exclusively on adult populations;
- They were editorial comments, letters to the editor or conference abstracts;
- Neither the topic of motivation nor the use of digital technology in weight loss among children and adolescents was directly addressed.

Publications focusing solely on dietary, psychotherapeutic or educational aspects without including a digital technology component (e.g., apps, mobile devices, online interfaces) were excluded. However, studies examining the impact of social media were included, provided that their potential impact on motivation, health behaviour, body image or psychological well-being—factors relevant to the prevention and treatment of obesity—was assessed.

The decision to include only studies focusing on digital technologies was aimed to obtain consistent and comparable results that reflect the potential of these modern tools to address the growing problem of obesity among young people.

### 2.3. Selection Process

In the first stage, two independent reviewers screened the titles and abstracts of all identified articles (*n* = 3005) for initial relevance, based on predefined inclusion and exclusion criteria. Discrepancies in decisions were resolved through discussion or consultation with a third reviewer.

In the second stage, full texts of the eligible studies (*n* = 225) were retrieved and assessed in detail for methodological quality and relevance. Studies were excluded if they lacked behavioural outcomes, did not involve digital technologies, or failed to target the population of children and adolescents. Full-text review resulted in the exclusion of 47 articles, with 59 meeting all criteria.

**Figure 2:**
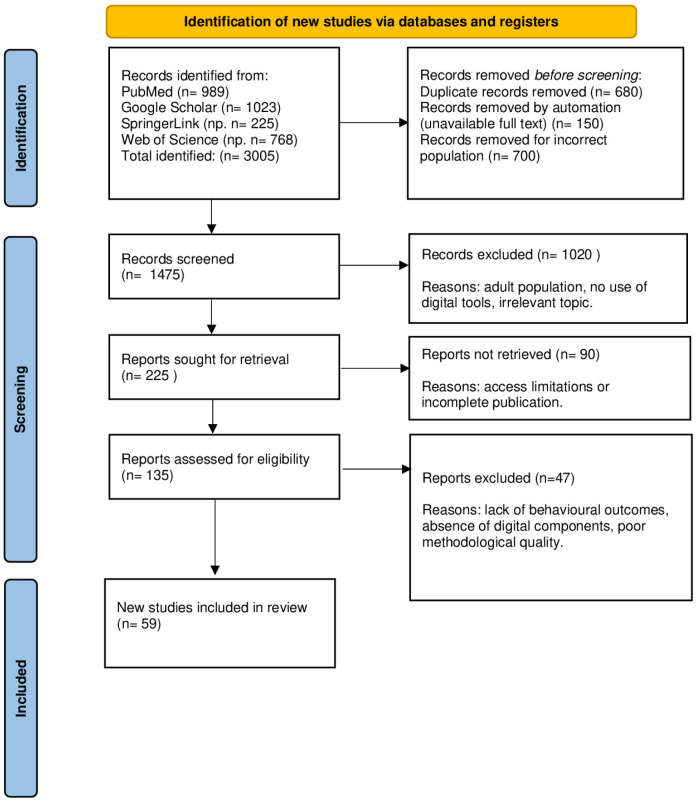
The PRISMA literature search and selection process.

Reasons for exclusion at each stage of the process (e.g., lack of digital component, adult population, editorial format) are detailed in the PRISMA flow diagram (Figure 2). No automation tools were used in the screening process.

Four databases were searched: PubMed (*n* = 989), Google Scholar (*n* = 1023), Springer Link (*n* = 225) and Web of Science (*n* = 768). A total of 3005 publications were identified. Prior to the screening stage, 680 duplicates were removed, 150 publications were automatically rejected due to the lack of a full text, and 700 were excluded due to the inappropriateness of the study population (e.g., adult studies), or the lack of a digital component. A total of 1475 publications were shortlisted for the initial screening stage, of which 1020 were rejected after analysis of titles and abstracts. Subsequently, 225 publications were referred for full-text analysis, but 90 could not be retrieved due to limited access. The remaining 135 full-text articles were assessed for compliance with the inclusion criteria. After further analysis, 47 publications were excluded, mainly due to a lack of behavioural outcomes, a lack of digital technologies or poor methodological quality. Ultimately, 59 studies were included in the final review.

## 3. Results

Following the literature review, a total of 59 scientific publications that met the predefined inclusion criteria were included in the analysis. These papers focused on cutting-edge technological interventions to support children and adolescents to reduce weight, improve lifestyle and increase motivation to engage in healthy behaviours. In order to organise the collected material, the results were grouped thematically according to the type of technological intervention and the main health effects reported in the studies.

### 3.1. Personalisation and the Role of the Family in Digital Technologies Interventions

Studies assessing the role of digital technologies in the context of personalising interventions and family involvement in the treatment of obesity in children and adolescents were reviewed. Many of the publications reviewed highlight that the personalisation of the message and caregiver participation significantly increase the effectiveness of health interventions [12,13]. Interventions based on mobile apps, online platforms and smart devices show positive effects on weight management, especially when they combine education, behavioural monitoring and contact with caregivers [15,16]. It has also been pointed out that, while technology alone can facilitate the implementation of health changes, it does not achieve the intended results without adequate support from the family and an educational environment. Importantly, the long-term engagement of users, including adolescents, is enhanced through the personalisation of the message, motivational features and feedback [17]. Although not all studies have shown a statistically significant reduction in BMI, many note positive behavioural changes, such as improved eating habits and increased physical activity [18,19,20,21] (Table 1).

### 3.2. Mobile Apps and DTs in the Treatment of Obesity

The results of studies evaluating the effectiveness of mobile apps, interactive features and digital technologies to support obesity prevention and treatment in children and adolescents were analysed. The analysed publications indicate that these technologies can effectively support health behaviour change, especially when they are personalised, provide ongoing feedback and engage the user through gamification, progress monitoring or educational elements [23,24,25,26,27,28,29,30,31,32]. Similar conclusions were drawn in the meta-analysis by Kouvari et al., where digital interventions produced significant BMI reduction effects primarily in children with a markedly elevated BMI, suggesting the potentially greater effectiveness of such measures in participants with obesity [13]. Other studies have shown that adolescents with obesity (BMI ≥ 97th percentile) achieved greater weight loss than those who were overweight, indicating a higher effectiveness of digital interventions in groups with higher baseline BMI values [31]. Interventions combining applications with specialist counselling and family support proved to be particularly relevant, confirming the high effectiveness of comprehensive approaches. Despite the generally positive results, individual studies highlight the need to further adaptation of the tools to meet the specific needs of the target audience (Table 2).

### 3.3. SMS and Mobile App Limitations

Studies evaluating the effectiveness of short text message (SMS)-based technological interventions and the limitations of mobile apps in the prevention and treatment of obesity among children and adolescents were also included in the analysis. Short text messages, according to parents, are easy to learn and can effectively support children’s daily health decisions [33]. However, the results of intervention studies are mixed, with one showing no significant changes in BMI and psychological well-being after SMS Maintenance Treatment (SMSMT. In contrast, other reviews show a moderate effectiveness of mobile apps and e-learning platforms in improving health and psychosocial behaviours in adolescents. Despite an initial increase in motivation associated with wearable technologies (e.g., Fitbit), the effect was short-lived mainly due to social pressure and competition [34]. Importantly, interventions in culturally specific groups—such as among adolescents of Hispanic origin—still need better evaluation of feasibility and long-term effectiveness [35] (Table 3).

### 3.4. Socioeconomic Factors Affecting Access to Digital Intervention

Studies on the effectiveness of digital health interventions have shown that individuals with a lower socio-economic status (SES) have limited access to eHealth and lower digital literacy, which negatively impacts the effectiveness of such programmes. Income remains significantly associated with the ability to use digital tools, and universal interventions often fail to meet the needs of this group. Therefore, when developing digital health strategies, it is essential to consider technological, financial, and social barriers to avoid exacerbating existing inequalities. Moreover, research has shown that only a small proportion of participants with a low SES achieved clinically significant weight loss (≥5%), and one study did not meet national guidelines for intervention effectiveness. These findings highlight the need for more tailored and effective solutions for this population [36,37,38,39] (Table 4).

### 3.5. Machine Learning, Educational Games and Robotics in the Treatment of Obesity

A number of studies are devoted to the use of digital technologies—including the Internet of Things (IoT), mHealth applications, machine learning and interactive digital tools—in the prevention and treatment of obesity among children and adolescents. Two studies have shown a positive impact of technology on lifestyle change, including improved physical activity, healthy eating and motivation to maintain a healthy body weight [40,41]. Interventions engaging users through gamification, the personalisation of the message and the co-creation of tools with children and their carers have proven particularly effective. One study highlights the effectiveness of mobile applications based on psychological behaviour models such as the Technology Acceptance Model (TAM), the Theory of Planned Behaviour (TPB), the Theory of Meaningful Behaviour (TMB)) and the Big Five personality trait concept. These applications, as demonstrated in the study, can effectively motivate teenagers to engage in physical activity and support the formation of lasting pro-health habits. However, it is worth noting that not all forms of digital technologies bring unequivocal benefits—if used inappropriately, they can encourage a sedentary lifestyle and further exacerbate the problem [42] (Table 5).

### 3.6. Technology’s Affects on Body Image and Mental Health

The influence of digital technologies on the body image and the mental health of children and adolescents is a topic that is increasingly being addressed in the scientific literature. Systematic reviews and empirical studies show that social media and other forms of digital presence significantly influence the way teenagers perceive their own bodies. The work of Aubrey et al. indicates the significant impact of phenomena such as selfies, photo retouching and fitspiration on increasing appearance pressure. Similar conclusions result from the review by Holland and Tiggemann, where as many as 20 analysed studies indicate the negative impact of using social networking sites (SNSs) on body image and the risk of developing eating disorders [44]. On the other hand, the qualitative study by Papageorgiou et al. shows that girls aged 14–17 experience increased social comparisons, leading to low body satisfaction and increased pressure to modify their appearance [45]. Narrative and theoretical reviews, on the other hand, suggest the need to design digital solutions that not only minimise the risk of worsening mental health, but can also support positive body image [46] (Table 6).

## 4. Discussion

Currently, obesity is a growing public health problem faced by specialists across a variety of fields, including doctors, nutritionists, psychologists, as well as educational and family environments. Due to the rapidly growing number of cases of obesity among children and young people, educational institutions and the media should promptly implement prevention programmes and information campaigns to curb the ever-increasing incidence rate of obesity. The World Health Organisation has indicated that the global scale of obesity has almost tripled since 1975 [1].

This trend is driven by an ongoing advancement of modern technologies, which profoundly shape our daily life—now increasingly characterised by mechanisation, computerisation and automation. As a result, everyday activities that used to require physical activity are now being replaced by intelligent systems that reduce the daily dose of exercise to a minimum [49]. In addition, children and young people increasingly spend their free time on the internet, social media, playing computer games and watching television, which further reduces their level of physical activity. Numerous studies confirm the link between the intensive use of digital media and the risk of obesity [50]. In addition, a lack of control over the time spent in front of a screen can lead to delays in physical development and a reduction in children’s social and cognitive abilities. Nonetheless, digital technologies can effectively support the promotion of a healthy lifestyle and healthy eating habits [51].

Excessive body weight in young people is associated with reduced quality of life, low self-esteem, difficulties in peer relationships and an increased risk of depressive symptoms. The use of health apps can also positively influence the mental well-being of children and adolescents. One of the primary goals of digital technology is to encourage regular physical activity among young people. Numerous studies confirm that physical exercise has a significant impact on reducing major depressive disorder symptoms. The most substantial benefits are seen in aerobic training sessions lasting 40 to 50 min, carried out three times a week for at least 12 weeks [52,53,54].

Therefore, the aim of this study is to analyse publications on the impact of digital technologies on shaping the motivational attitude toward weight loss among children and school-age youth. Additionally, in our study, we focused on assessing the utility of these solutions in the context of reducing the BMI and their role in the prevention of obesity in the young population.

### 4.1. Key Factors Influencing the Effectiveness of Digital Interventions

Currently, a wide range of applications is available, aiming to motivate users to improve their lifestyle. As the results of research by Altıntas Basar et al. indicates, digital technologies enable not only access to expert knowledge, but also the monitoring and analysis of food consumed, which facilitates the observation of changes taking place in the body [12]. Digital technologies can serve as an effective adjunct to traditional approaches in the treatment of obesity among children and adolescents. It is noteworthy that mobile applications and fitness bands yield particularly positive effects when supported by the active and conscious involvement of parents [13,17,18,19].

An important factor in shaping healthy eating habits is the provision of adequate health education and early awareness-raising among young people about the principles of healthy lifestyle. In their study, Lazorick et al. implemented the MATCH programme in primary schools, which consisted of an integrated educational approach combining traditional learning with elements of technology to support lifestyle change. The long-term results are promising: the programme participants gained significantly less weight than their peers who did not participate in the programme. This study shows that early health education supported by technology can shape long-lasting healthy habits [15]. Moreover,, it is essential that digital technologies used in obesity prevention are tailored to the cognitive development level of young users [17]. Unfortunately, many applications focus mainly on functionality and aesthetics, which can limit their long-term effectiveness [20]. Despite the growing popularity of digital applications, research demonstrates that they cannot replace direct contact with another person. The best results are observed when technologies are used in parallel with the care of a specialist or trainer [21,22].

The effectiveness of digital interventions may depend on participants’ baseline body weight status, as confirmed by data from a study involving 2825 Chinese adolescents with overweight or obesity (BMI ≥ 85th percentile). After 120 days of a fully remote intervention combining a mobile app, self-weighing, and calorie control, the average weight loss was 8.6 ± 0.63 kg, and the BMI reduction was 3.13 ± 0.21 kg/m^2^. As many as 71.4% of participants achieved a ≥5% reduction in body weight, with greater effectiveness observed among those with a BMI ≥ 97th percentile and among girls, who showed a significantly greater decrease in BMI z-score compared to boys (*p* < 0.004). These results indicate that individuals with a higher baseline BMI benefit more from participating in digital health programmes [33]. Meanwhile, a meta-analysis of eight randomised clinical trials involving 582 children and adolescents demonstrated that technology-based interventions can effectively support weight reduction, especially when used as an adjunct to standard care. Significant BMI-related changes were reported in five of the eight studies, particularly among children with obesity [13].

In a six-week study by Edwards et al., 18 teenagers used two types of pedometers and a shared online platform for data recording and social interaction. The researchers noted that the ability to share their results with their peers can motivate children and young people, thus encouraging them to be more physically active [23].

The main purpose of digital technology tools is to support, monitor progress and increase the effectiveness of pre-planned weight loss programmes [24]. Internal motivation can be strengthened by using features such as real-time feedback, reward systems or the ability to track progress. For this reason, so-called ‘just-in-time’ interfaces are increasingly used, which enable the dynamic adaptation of health messages to the current behaviour of the user [26]. The most effective applications are those that integrate personalisation, real-time feedback and professional advice, allowing for a more personalised intervention [25]. Gilmore et al. considered a wide range of mobile applications such as MyFitnessPal, Nike Training Club and FitBit, which differ in their range of functions—from monitoring physical activity and nutrition to motivational elements, gamification and the personalisation of goals. Apps such as Couch to 5K and Map My Run enable users to gradually increase their physical activity, while Fooducate and My Meal Mate support better food choices. Thanks to progress tracking, data reporting and phone integration, app users can monitor their progress and specialists can monitor the patient’s health on an ongoing basis [27].

Some studies point to the significant impact of cultural and ethnic factors on the effectiveness of mobile health technologies in reducing BMI in children and adolescents with obesity [28]. An example is the PEGASO Fit for Future (F4F) project—a mobile health application co-created by 74 teenagers from Spain, Italy and the UK. The system, which combines self-monitoring, counselling, entertainment and social support functions, has been adapted to the preferences of young people from different cultural backgrounds. This was the first study to show that a complex mHealth system can be designed in collaboration with young people from different countries [29]. In their study, Hagman et al., used a digital support system with wireless scales connected to an application, along with continued communication with medical staff. In the intervention group (107 children and their families), individually tailored feedback was used, and produced better results than standard care in the control group (321 children) [30]. An interesting platform supporting the treatment of childhood obesity is ETIOBE—an eHealth system comprising three integrated components: the Clinician Support System (CSS) which assists doctors, the Home Support System (HSS) which engages the family, and the Mobile Support System (MSS), a mobile application that facilitates children’s daily self-monitoring and habit formation. An interesting result was obtained in a study by Lei et al. in which 2825 children aged 10–17 used a mobile application connected to a wireless scale. The nutritional programme was tailored to the individual metabolic needs of the participants (BMR according to the Schofield equation). After 120 days, as many as 70% of the children had achieved a weight reduction of ≥5% [31]. Although not all studies show a significant reduction in BMI, as in the case of the OBEST app, positive changes in eating behaviour, such as a reduction in the consumption of fast food, have been observed [32].

Another important factor influencing the effectiveness of digital technology interventions is the SES of the target population. Studies have emphasised that individuals with a lower SES often have significantly reduced access to eHealth tools and lower levels of digital literacy, which negatively impacts their engagement and outcomes [55]. Despite the growing popularity of eHealth interventions for promoting healthy lifestyles, their effectiveness among low SES individuals faces major barriers related to technology, finances, and limited user engagement. Research shows that universal digital programmes may be insufficiently tailored to the needs of this group, highlighting the need to design more flexible and customised solutions that include, for example, social support, offline access, and simplified user interfaces [36,56]. Kristjánsdóttir et al. conducted a study in Sweden to determine how socio-demographic factors affect eHealth literacy among parents of children requiring paediatric surgery. In this study, 30 parents completed the eHealth Literacy Questionnaire along with a survey on education, income, age, gender, and place of residence. Among the variables analysed, monthly income showed the strongest and most positive correlation with eHealth literacy levels across all seven domains assessed. These findings suggest that economic status—more than other demographic factors—plays a pivotal role in shaping individual’s ability to use eHealth tools, which is essential for ensuring equitable access to technology-supported healthcare [37,38]. Other authors also note that eHealth literacy is closely linked to SES—those in less-privileged socio-economic positions typically demonstrate lower skills in this area [57]. This was demonstrated in a pilot study involving 55 low SES adults with overweight or obesity, who participated in a 12-week weight loss intervention based on social media (Facebook group + Fitbit). Participants who completed the programme (*n* = 47) lost an average of 1.07 kg (*p* = 0.0498), and 96% said they would recommend the programme to others. These results indicate that such an intervention format is feasible and well-received in low SES groups [39]. Another study involving 104 low-income women (84% from rural areas) found that a text message–based programme effectively increased physical activity, step count, and supported weight loss. The results confirm that such interventions can be effective in resource-limited populations [36]. Similarly, Griffin’s 12-week study among 104 low-income, rural women reported significant improvements in dietary behaviour, physical activity, and weight reduction after receiving daily health-related text messages. Participants were more likely to set health goals and monitor their own progress [58].

One of the simpler but equally effective forms of technological support in health interventions is SMS messaging. Regular reminders, motivational messages and short tips sent directly to the user’s phone can strengthen engagement and help maintaining healthy habits. The results of the Sharifi study conducted on a group of 31 parents of overweight and obese children show that even simple technology such as SMS messaging can be an effective tool supporting health behaviour change [33].

Furthermore, studies on the use of devices such as Fitbits or other trackers have shown that the technology can sometimes reduce motivation, causing pressure and frustration, especially in children aged 13–14 [34]. Therefore, to maintain the long-term effects of the therapy, it is necessary to combine technology with traditional forms of support—including health education, contact with a guardian and community activities.

A growing body of research indicates that game-based activities are an effective form of support in reducing obesity, especially among children and young people. Research has shown that these types of digital solutions can be more engaging and effective than traditional health apps [40]. An example of this approach is the pilot study by Zarkogianni et al., who presented the ENDORSE platform—an application integrating mHealth technologies, artificial intelligence (AI) and serious games to support the management of childhood obesity. In a three-month intervention involving 50 children, a clinically significant decrease in BMI was achieved [43]. The combination of gamification, motivational approaches and health education can influence changes in health behaviour at an early stage of life [41].

The younger generation is characterised by a high level of digital competence, making it an ideal target group for mobile app-based interventions. Research shows that digital solutions combining physical activity, technology and elements of gamification effectively support the development of positive health attitudes and promote weight loss. This is confirmed by the results of a study by Bastida et al., who implemented an intervention among children aged 9–12 using the OCARIoT application. Thanks to personalised educational missions, support from mentors and the use of a reward system, the application significantly increased the participants’ engagement, leading to a reduction in the obesity rate by as much as 75.5% in the intervention group [42].

Digital technologies, particularly social media, play a significant role in shaping body image and self-esteem among children and adolescents. A growing body of research indicates that intensive use of these platforms can negatively impact the perception of one’s own appearance and contribute to lower self-esteem [47]. In their literature review Aubrey et al. emphasise the importance of sociocultural theories of social comparison in the context of adolescents’ use of digital technologies. The authors analyse factors such as selfies, photo retouching, fitspiration, online forums and general presence in social media as potential contributors to distorted body image among young people [44]. According to the study findings, platforms such as TikTok can significantly impact the behaviour and mental health of young people. Their mechanisms—driven by a system of rewards, likes and social interactions—stimulate the dopaminergic system. Their use has also been associated with a sedentary lifestyle, reduced sleep quality, cognitive and emotional disorders, and lower academic performance [46]. In a systematic review of 20 studies, Holland and Tiggemann demonstrated a clear association between the use of SNSs and body dissatisfaction, as well as symptoms of eating disorders. Activities such as viewing and posting photos proved to be particularly problematic [48]. Moreover, other studies indicate that teenage girls are particularly vulnerable to the influence of social media on their body image perception [45].

Taking the above into account, it is important to note that a distorted body image and reduced psychological well-being can significantly affect the internal motivation of children and adolescents to engage in healthy activities, including weight reduction. Therefore, the design of digital interventions supporting weight loss should consider not only behavioural but also psychological factors, in order to minimise the risk of exacerbating negative emotions while simultaneously fostering a positive relationship with one’s own body.

### 4.2. Clinical and Practical Implications

From a clinical perspective, the findings of this review highlight the potential of digital technologies to complement traditional weight management strategies among children and adolescents. Mobile applications, wearable devices, and gamified platforms can provide personalised feedback, increase motivation, and improve adherence to healthy behaviours, particularly in populations that are difficult to engage through conventional methods.

These tools may be especially useful in school-based programmes, primary care settings, or family interventions, where ongoing monitoring and interactive engagement are key. For healthcare professionals, the integration of digital technologies into obesity prevention and treatment plans may provide scalable and cost-effective means of supporting behaviour change and sustaining long-term outcomes.

### 4.3. Limitations

Several limitations are evident in the analysed publications. These include methodological differences between the analysed publications in intervention duration, participants age, and the types of technologies employed. Moreover, most studies did not include comparisons between participants with obesity and those with normal body weight, which limits the ability to assess the extent to which baseline BMI influences the effectiveness of digital technology interventions. Additionally, many studies were short-term, making it difficult to evaluate the durability of the effects of using digital technologies in the treatment and prevention of obesity. The literature lacks data on the sustained maintenance of reduced body weight, lifestyle changes, and the impact on mental health following the conclusion of the intervention. A further limitation is that some of the analysed publications were based on small sample size or lacked a control group, which makes it difficult to assess the actual effectiveness of the technological solutions used. An additional limitation is the lack of information, in some studies, regarding the cognitive development level of children and adolescents, which is important in the context of designing appropriate interfaces and educational content. Cultural differences were also not always adequately considered, despite their potential to significantly affect the effectiveness of technological interventions. Therefore, further and more standardised research, grounded in robust methodology, is needed across diverse populations and settings, taking into account both the short- and long-term effects of the use of digital technologies in the context of childhood and adolescent obesity.

## 5. Conclusions

Based on the literature review, digital technologies appear to be a promising tool in addressing the growing problem of obesity among children and school-aged youth. Mobile applications, educational games, activity trackers, mHealth platforms and artificial intelligence-based systems can support both the treatment and prevention of obesity. Notably, interventions that incorporate personalisation, feedback and social support tend to be the most effective, particularly when they involve parents or caregivers.

Importantly, the technologies analysed not only contribute to BMI reduction but also positively impact the mental health of children and young people, supporting the development of a positive body image, self-efficacy and self-esteem. Features such as progress tracking, gamification and health education enhance engagement in lifestyle change processes, thereby increasing the sustainability of outcomes achieved.

It is important to emphasise that digital technologies alone are not sufficient; their effectiveness depends on both the context of implementation and the level of young user engagement. Inadequately designed digital technologies carry the risk of contributing to a deterioration in well-being, increased social pressure, or the reinforcement of negative comparison patterns. To address this challenge effectively, future strategies to combat obesity should use technology to support of multidisciplinary initiatives combining medical, psychological, pedagogical and technological knowledge. Developing guidelines for the design and implementation of digital health tools for children and adolescents is also essential, with careful consideration of their cognitive, emotional and social development.

## Figures and Tables

**Table 1:** Personalisation and the role of the family in DT interventions.

Researchers	Aim	Materials and Methods	Results
Altıntaş Başar & Bilici (2023) [12]	To evaluate the impact of digital technologies (DTs) on nutrition education in prevention, with focus on children and adolescents.	Narrative review: Application of mobile apps, web-based interventions, personal digital assistive technologies, interactive computer-based technologies, photo- and video- based tools, wearables, distance education technology and (AI), in nutrition education.	DTs enhance engagement, support nutrition education through easy monitoring, data recording and personalisation.
Kouvari et al. (2022) [13]	To evaluate the effectiveness of technology-based interventions for the treatment of obesity in children and adolescents.	Systematic review and meta-analysis (2000–2021): Application of mobile apps, web platforms and SMS. Included 9 articles (8 studies).	Significant ^1^ BMI reduction (SMD = −0.61, *p* = 0.01); effect lost without parental involvement (SMD = −0.36, *p* = 0.14).
Lazorick et al., 2014 [15]	To evaluate the impact of an educational and technological programme on the prevention of obesity in young people.	Longitudinal study: (N = 106). Application of the ^2^ MATCH programme (health-themed lessons, behavioural strategies, web-based resources). Intervention group.	Significant decreases in ^1^ BMI z-scores in the intervention group (*p* = 0.002; reduction of 20–12% in weight). At 5-year follow-up, 2% of intervention group and 13% of comparison group had become overweight (*p* = 0.02).
Schoeppe et al. (2016) [16]	To assess the efficacy of app-based interventions for improving diet, physical activity and sedentary behaviour.	Systematic review (2006–2016); 27 studies (70% ^3^ RCTs); apps used alone or in multi-component programmes.	Apps showed modest effectiveness, especially in multi-component interventions. Higher app usage correlated with better outcomes.
Kaakinen et al., 2018 [17]	To evaluate technology-based counselling to support children and adolescents who are overweight or obese.	Descriptive systematic review (until 2015). Application of e-counselling, interactive multimedia games, CD ROMs, SMS, TV and emails. Included 28 articles (14 ^3^ RCTs).	No statistically significant changes in ^1^ BMI; some studies noted improvements in fruit and vegetable intake and activity.
Quelly et al. (2016) [18]	To evaluate the impact of mobile apps on anthropometric, psychosocial and behavioural indicators associated with obesity in children and adolescents.	Qualitative and quantitative systematic review: Application of mobile apps included 9 articles.	Mixed results: positive effects on motivation and goal-setting behaviours, inconclusive impact on physical activity, dietary habits, and anthropometric measures.
Fowler et al. (2021) [19]	To evaluate of the effectiveness of DT interventions in the treatment and prevention of obesity in children and adolescents.	Systematic review and meta-analysis: Application of mobile apps, web-based programmes, SMS, telemedicine, exergaming, wearables. Included 55 ^3^ RCTs.	Small but significant effect on weight loss (d = −0.13, *p* = 0.001); no effect of prevention interventions (d = 0.05, *p* = 0.52).
Bardus et al., 2015 [20]	To evaluate current evidence on the use of mobile and Web 2.0 technologies in prevention.	Systematic scoping review (2004–2014). Use of DTs for promoting behavioural change or measuring behaviour. Included 457 articles.	Mobile technology and Web 2.0 solutions have increasing potential as tools to support change.
Holmes et al., 2018 [21]	To evaluate of the effectiveness of digital communication technologies on weight loss maintenance.	Systematic review (2006–February 2018). Application of SMS, email and web-based system. Included 7 ^3^ RCTs.	Four ^3^ RCTs reported significant effect on weight loss maintenance in the short term of 3–24 months. One study (in children) found no significant difference in ^1^ BMI.
Dobbie et al., 2021 [22]	To evaluate the impact of eHealth interventions on physical activity (PA) and weight reduction.	Narrative review. Application of wearables, phone apps, SMS, and exergaming.	Wearable devices can increase PA and lead to moderate weight loss in middle/older children in the short term (<1). Data for mobile phone, ^4^ SMS, and exergaming interventions are less robust.

Abbreviations: ^1^ BMI—body mass index; ^2^ MATCH—Motivating Adolescents with Technology to CHOOSE Health; ^3^ RCT—randomised clinical trial; ^4^ SMS—short message service.

**Table 2:** Mobile apps and DTs in the treatment of obesity.

Researchers	Aim	Materials and Methods	Results
Kouvari et al., 2022 [13]	To assess the effectiveness of technology-based interventions for obesity treatment in children and adolescents.	Systematic review and meta-analysis (2000–2021); 8 ^7^ RCTs, 582 participants; most combined tech tools with standard care.	Significant ^3^ BMI reduction (^9^ SMD = −0.61, *p* = 0.01); effect not significant without parental involvement (^9^ SMD = −0.36, *p* = 0.01).
Edwards et al., 2011 [23]	To evaluate teenagers’ motivation for physical activity (PA) and their expectations of probe-type technology.	Qualitative study: (^1^ N = 12; 6-week); Application of use of two differentpedometers, shared online platform for data recording and interaction.	Technology acted as a motivator and helped to achieve the goals set during the down process.
Kim et al., 2022 [24]	To evaluate the effectiveness of information and communication technology (ICT) in the management of obesity and metabolic syndrome.	Application of mobile apps, telemedicine, wearables, web-based platforms, ^10^ SMS and AI.	ICT-based interventions are accessible, flexible and efficient.
Chen et al., 2020 [25]	To evaluate the effectiveness of DTs in modifying eating behaviour as a form of obesity prevention.	Systematic review: Application of personal digital assistants, web-based educational tools, video games and mobile apps. Included 15 articles meeting the ^6^ PRISMA guidelines.	Efficacy of DTs increased when combined with counselling and personalised feedback.
Sandri et al., 2019 [26]	An analysis of DTs supporting obesity prevention. Introduction to the ^11^ STOP project.	Application of wearable devices, chatbots, gamification, data fusion and machine learning.	Highlights the importance of these DTs in delivering personalised, supportive feedback to enhance the effectiveness of health messages and supporting healthy weight.
Gilmore et al., 2014 [27]	Discuss the role of digital technology in successful management programmes.	Narrative review: Application of mobile apps, web-based interventions and ^10^ SMS.	Digital technologies enable improved long-term weight management and are cost-effective.
Yien et al., 2021 [28]	To evaluate the effectiveness of mobile health technologies in weight management in obese children.	Systematic review and meta-analysis: Application of mobile apps, telehealth counselling, web-linked activity trackers and gamification elements. Included 9 ^7^ RCT.	The intervention showed no significant effect in reducing ^3^ BMI (−0.773; 95% CI: −1.069 to −0.476).
Martin et al., 2020 [29]	To develop components of an mHealth system to promote healthy lifestyles in adolescents through co-design with users from three countries.	Iterative Co-Design and Feasibility Study: (^1^ N = 74; 13–16 years old in Spain, Italy and UK) Application of ^4^ PEGASO F4F mobile apps prototypes.	^4^ PEGASO F4F app should be personalised, age-appropriate, easy to use, goal-oriented, include rewards, and support peer connection.
Hagman et al., 2022 [30]	To evaluate the effectiveness of mHealth digital support system as adjunct to standard care in the treatment of obesity.	Interactive digital: (^2^ AG = 107; ^8^ SG = 321; 4.0–17.9 years old in Sweden). Application of Evira AB app.	At year one, the ^2^ AG showed a greater reduction in ^3^ BMI than the ^8^ SG (*Z*-score = −0.15, *p* = 0.012).
Lei et al., 2021 [31]	To evaluate the effectiveness of a mobile app weight loss programme in overweight children and adolescents.	Observational study: (^1^ N = 2825, 10–17 years old in China). Application of MetaWell mobile app.	After 120 days, a reduction in weight (^5^ *M* = −8.6 kg, *p* < 0.001) and ^3^ BMI (^5^ *M* = −8.6 kg, *p* < 0.001; *Z*-score = −0.42, *p* < 0.001) was observed.
Likhitweerawong et al., 2021 [32]	To evaluate the effectiveness of a mobile app as a tool to support weight reduction and to promote healthy eating habits in children.	Randomised controlled trial: (AC = 35, ^8^ SG = 35 in Thailand). Application of OBEST mobile app.	No significant between group differences in weight, ^3^ BMI change or *Z*-score and waist circumference.
Lei et al., 2021 [31]	To assess the effectiveness of a fully remote digital weight loss programme in adolescents with overweight/obesity.	Observational study; 2825 adolescents (10–17 yrs) in China used a programme combining mobile apps, self-weighing, calorie restriction, and meal replacement.	Significant reductions in weight, ^3^ BMI, and ^3^ BMI z-score by day 120. Higher app use and ^3^ BMI at baseline were linked to greater weight loss. Girls showed better ^3^ BMI z-score reductions than boys.

Abbreviations: ^1^ N = number; ^2^ AG—active-care group; ^3^ BMI—body mass index; ^4^ PEGASO F4F—Personalised Guidance Services for Optimising lifestyle management in teenagers through awareness, motivation, and engagement in Fit for Future; ^5^ M = mean; ^6^ PRISMA—Preferred Reporting Items for Systematic Reviews and Meta-Analyses; ^7^ RCT—randomised clinical trial; ^8^ SG—standard-care group; ^9^ SMD—standardised mean difference; ^10^ SMS—short message service; ^11^ STOP—Science and Technology in childhood Obesity Policy.

**Table 3:** SMS and mobile app limitations.

Researchers	Aim	Materials and Methods	Results
Sharif et al., 2013 [33]	To evaluate parental acceptability and preferences for using SMS and mobile technologies to support obesity-related behaviour change.	Qualitative study: Application of SMS. Five focus groups (parents ^1^ N = 31 of overweight or obese children 6–12 years old).	Parents viewed SMSs as convenient; preferred their child’s doctor-endorsed, actionable strategies sent two to three times weekly.
Kerner & Goodyear, 2017 [34]	To evaluate if healthy lifestyle wearable technologies (e.g., Fitbit) impact teenagers’ motivation to be active.	Mix methods study: (^1^ N = 84, 13–14 years old). Application of Fitbit. Eight-week intervention.	Significant impact on motivational outcome post-treatment across time (F_(6, 77)_ = 8.72, *p =* 0.00, η = 0.41.Declines in competence (F = 8.5, *p* = 0.005, η^2^ = 0.91), autonomy (F = 13.49, *p* = 0.00, η^2^ = 0.14), relatedness (F = 5.81, *p* = 0.02, η^2^ = 0.07), autonomous motivation (F = 17.00, *p* = 0.00, η^2^ = 0.17). Increase in amotivation (F = 38.00, *p* = 0.00, η^2^ = 0.32). Short-term motivation observed due to novelty, with engagement dropping after 4 weeks.
Soletro et al., 2022 [35]	To evaluate sample and intervention of DT-based interventions to prevent obesity among Hispanic adolescents in the USA.	Scoping review. Application: web-based session, wearable devices (e.g., pedometer), ^3^ SMS, video gaming. Included 7 ^2^ RCTs.	The review showed that although there are promising technological interventions among Hispanic adolescents, there is still a lack of robust research on their feasibility and effectiveness.

Abbreviations: ^1^ N = number; ^2^ RCT—randomised clinical trial; ^3^ SMS—short message service.

**Table 4:** Studies addressing the influence of socioeconomic factors on the effectiveness and accessibility of digital health interventions.

Researchers	Aim	Materials and Methods	Results
Griffin et al., 2018 [36]	To assess the effect of a 12-week text messaging programme (My Quest) on dietary habits, physical activity, and weight in low-income women.	One-group pre-post design; 104 women from 55 Alabama counties (84% rural), mostly overweight/obese. Intervention included 2–3 daily texts with health tips and goal-setting prompts, weekly e-newsletters, and weekly self-weighing.	Statistically significant improvements (*p* < 0.05) in dietary and physical activity behaviours, goal setting, and food environment. Body weight was significantly reduced (no exact kg reported). Intervention was low-cost and feasible for rural, low-income settings.
Kristjánsdóttir et al., 2023 [37]	To assess eHealth literacy domains in parents of children needing paediatric surgery and their correlation with socioeconomic and demographic factors.	Descriptive correlational study; 35 parents in Sweden (30 completed full questionnaires); part of a larger clinical trial. Assessed 7 eHealth literacy domains and 5 socioeconomic/demographic variables.	Parents showed strengths in digital skills, control, and safety; weaknesses in motivation and platform accessibility. Overall literacy was adequate. Monthly income had the strongest positive correlation with eHealth literacy domains.
El Benny et al., 2021 [38]	To examine how digital health interventions (DHIs) assess domains of the eHealth literacy model, and which technologies and health issues they address.	Scoping review of 131 ^1^ RCT-based DHIs (2001–2020) from 26 countries. Sources: MEDLINE, CINAHL, Embase, Cochrane. Data charted by country, health condition, technology used, and literacy domains.	61.8% of DHIs were from English-speaking countries; 51.9% were web-based; 43.5% targeted NCDs, 19.8% mental health. 73.2% assessed health literacy, 14.5% digital literacy, 3% basic/media literacy, 0.7% scientific/information literacy. None covered all six domains. Only 5.3% assessed both health and digital literacy.
Cavallo et al., 2021 [39]	To evaluate the feasibility of a 12-week social media–based weight loss intervention for low socio-economic status (SES) overweight/obese adults.	One-group pre-test-post-test pilot; 2 cohorts (*n* = 39, *n* = 16; total *n* = 55); participants used Fitbits and engaged in a private Facebook group moderated with educational content and support.	High retention (86%); 9175 interactions recorded. 96% of completers (*n* = 47) would recommend the programme. Mean weight loss: 1.07 kg (^2^ SD = 3.96, *p* = 0.0498); increase in dietary social support (mean = 2.47, ^2^ SD = 5.09, *p* = 0.0007). High engagement suggests feasibility for low SES populations.

Abbreviations: ^1^ RCT—randomised clinical trial; ^2^ SD—standard deviation.

**Table 5:** Machine learning, educational games and robotics in the treatment of obesity.

Researchers	Aim	Materials and Methods	Results
Lam et al., 2022 [40]	To evaluate the use of Internet of Things (IoT) enabled technologies in interventions to reduce childhood obesity.	Systematic review (2010–2019): Application of IoT architecture (sensory, network, service, application layers). Included 23 articles meeting the ^6^ PRISMA guidelines.	Devices: smartphones/apps (78.3%), accelerometers (56.5%), smartwatches (Fitbit in 4 studies). Data types: physical activity ([PA] 65.2%), diet, sleep. Techniques: monitoring (91%), feedback (45%), goal setting (31.8%), gamification (31.8%). Game-based interventions showed better engagement.
Vlachopapadopoulou et al., 2019 [41]	Overview of mHealth solutions for the treatment and monitoring of childhood obesity.	Discussion of the latest mHealth technologies used for self-treatment and health support for children with obesity.	mHealth technologies can support behavioural change and play an important role in weight management for children and adolescents.
Bastida et al., 2023 [42]	Development of the ^5^ OCARIoT platform promoting obesity prevention to support healthy habits in children.	Mixed methods pilot study (Phase 1 prototyping N = 334, Phase 2 intervention N = 127 in 4 schools in Spain, Greece and Brazil). Application of wearables (Fitbit), smart weight scale, ^4^ NFC, IoT air/environmental sensors, ^3^ DAP, ^2^ API Gateway, DSS, dashboard, gamified app.	Decrease in the prevalence of obesity by 75.5% in the intervention group compared to the baseline and decrease in the percentage of children with thinness to 1.33%.
Zarkogianni et al., 2023 [43]	To evaluate the impact of parenting styles and psychosocial factors on the effectiveness of the ENDORSE programme in reducing BMI in children.	Pilot study: (50 mothers and their children, 6–14 years old) Application of app, wearables (Fitbit), serious games and ^1^ AI. 3-month interventions.	Decreased ^7^ BMI (z-score −0.21, *p* < 0.001), fast food intake (−0.22 servings/week, *p* = 0.042), and screen time (−0.47 h/day, *p* = 0.005). Increased fruit (+0.62 servings/day, *p* < 0.001) and vegetables intake (+0.80 servings/day, *p* < 0.001), PA (+24.33 min/day, *p* < 0.001) and sleep (+0.54 h/day, *p* = 0.005).

Abbreviations: ^1^ AI—artificial intelligence; ^2^ API—application programming interface; ^3^ DAP—data acquisition app; ^4^ NFC—near filed communication; ^5^ OCARIoT—Smart Childhood Obesity Caring Solution using IoT Potential; ^6^ PRISMA—Preferred Reporting Items for Systemic Reviews and Meta-Analyses; ^7^ BMI—body mas index.

**Table 6:** Technology’s affects on body image and mental health.

Researchers	Aim	Materials and Methods	Results
Papageorgiou et al. [45]	Exploring the impact of sexualised images on social media on mental health and body perceptions in adolescent girls.	Qualitative study (N = 24, girls 14–17 years old in Australia).	Participants pointed out frequent appearance comparisons, deepening body dissatisfaction; social media increased the pressure to modify appearance.
Pedrouzo & Krynski, 2023 [46]	To evaluate the impact of TikTok use on young people’s physical, mental and behavioural health.	Narrative review (past five years).	TikTok influences behaviour patterns in young people and stimulates the dopaminergic reward system. Excessive use is linked to behavioural addictions, sleep disorders, obesity, decreased cognitive function and academic underperformance.
Demaria et al., 2024 [47]	To evaluate the impact of digital technologies (DTs) on adolescents’ body image perception and psychosocial development in the context of social media and culture.	Narrative review (June 2017–July 2024). Included 14 articles according to ^1^ PRISMA guidelines.	The expansion of DTs and social media reinforces a culture of appearance and the thin ideal, contributing to body dissatisfaction and a higher risk of developing eating disorders.
Holland & Tiggemann [48]	To evaluate the effect of social networking sites (SNSs) on the body and disordered eating.	Systematic review (until May 2015). Included 20 articles.	Using social networking sites is linked to negative body image and eating disorders. No effect of gender.

Abbreviations: ^1^ PRISMA—Preferred Reporting Items for Systematic Reviews and Meta-Analyses.

## Data Availability

All data and materials are included in the manuscript.
